# Comparison of Microbial Communities in Colorado Potato Beetles (*Leptinotarsa decemlineata* Say) Collected From Different Sources in China

**DOI:** 10.3389/fmicb.2021.639913

**Published:** 2021-03-19

**Authors:** Yanxue Yu, Yuhan Wang, Hongwei Li, Xin Yu, Wangpeng Shi, Junfeng Zhai

**Affiliations:** ^1^Institute of Plant Quarantine, Chinese Academy of Inspection and Quarantine, Beijing, China; ^2^Biowavelet Ltd., Chongqing, China; ^3^Department of Insects, School of Plant Protection, China Agricultural University, Beijing, China

**Keywords:** Colorado potato beetle (*Leptinotarsa decemlineata*), geographical sources, 16S rDNA, gut microbiota, biological invasion

## Abstract

Microbial communities in insects are related to their geographical sources and contribute to adaptation to the local habitat. The Colorado potato beetle (*Leptinotarsa decemlineata*) (CPB) is a potato pest that causes serious economic losses in Xinjiang Uygur Autonomous Region (XJ) and Heilongjiang Province (HL), China. The influence of microorganisms in the invasion and dispersal of CPB is unclear. We studied microbial communities of CPB collected from nine geographic sources in China using high throughput sequencing technology. Bacteroidetes, Firmicutes, and Proteobacteria were the most dominant phyla, Clostridia, Bacteroidetes, and γ-Proteobacteria were the most dominant classes, Enterobacterales, Lactobacillales, Clostridiales, and Bacteroidales were the most dominant orders, and Enterobacteriaceae, Streptococcidae, Verrucomicrobiaceae, and Rikenellaceae were the most dominant families. There were significant differences, among sources, in the relative abundance of taxa at the genus level. A total of 383 genera were identified, and the dominant bacteria at the genus level were compared between XJ and HL. *Pseudomonas* was the unique dominant microorganism in the HL area, and the other four microorganisms (*Lelliottia*, *Enterococcus*, *Enterobacter*, and *Lactococcus*) were common within the 2 regions. Bacterial community diversity in CPB from Urumqi, Jimunai, and Wenquan was higher than diversity in other regions. T-Distributed Stochastic Neighbor Embedding (tSNE) analysis indicated that order and genus were appropriate taxonomic levels to distinguish geographical sources of CPB. These findings provide insight into the diversity of microorganisms of CPB in the differences among geographically isolated populations.

## Introduction

Insects and microorganisms have evolved mutually beneficial relationships. Many microorganisms colonize the digestive tract and contribute to growth and development, nutrient metabolism, reproduction, immunity, pesticide resistance, and communication between hosts (Crotti et al., 2012). Extrinsic factors can affect the composition of the gut microbiota. Environmental factors and food type can also affect the composition of the gut microbiota. Honey bees from colonies in different habitats can have significantly different gut microbiota. The gut microbial community of bees foraging on rapeseed crops and bees not feeding on these crops differs (Jones et al., 2018). The composition and diversity of gut microbiota in different geographical populations of the Dark Gill Scarab (*Holotrichia parallela*) differ. This may be related to environmental factors, such as rainfall, temperature, and soil pH (Huang et al., 2012).

The Colorado potato beetle (CPB), *Leptinotarsa decemlineata* (Say), Coleoptera, Chrysomelidae) is a major pest of potatoes. It is native to the Rocky Mountains of North America and is now distributed worldwide (Hare, 1990; Alyokhin et al., 2008; Alyokhin, 2009). In China, CPB was first discovered in Xinjiang Uygur Autonomous Region in 1993 (Zhang, 1994; Wang et al., 2010). It is now found throughout Xinjiang Uygur Autonomous Region (XJ) and some areas of Heilongjiang Province (HL) where it causes serious economic losses (Guo et al., 2014, 2017).

Variable geographical conditions can divide insect distributions into different populations or ecological types. The differences among these geographical populations can involve ecological adaptability, resistance, and physiology (Tu et al., 2015; Xia et al., 2018). For example, the life history of the green peach aphid [*Myzus persicae* (Sulzer)] in China varies in different regions. *M. persicae* in the north is holometabolous, while in the south it displays incomplete metamorphosis, and both in the central region (Yang and Zhang, 1997). *Ostrinia furnacalis* does not diapause in the tropics, but the last seasonal generation of mature larvae diapauses during winter in temperate and subtropical regions (Tu et al., 2015). The adult weight and pupal weight of *Tribolium castaneum* gradually decrease from high latitudes to low latitudes. CPB develops faster in the short days than in the long days, and its pupa in the short-lighted areas is larger than that in the long-lighted areas (Dolezal et al., 2007). These examples demonstrate that the growth and development of insect species is related to their geographical location.

Host plant differences can influence gut bacterial communities and the symbiotic bacteria can help CPB adapt to host plants and can affect the interactions with other microorganisms such as nematodes (Michael et al., 2008; Chung et al., 2017). Gut microbiota may change within a relatively short time for species invading new areas (Berasategui et al., 2016), and gut microbiota can be used as a label for environmental conditions. However, there are no reports on the relationship between geographical sources and gut microbial diversity. Current studies of geographical sources mainly use genetic tests such as DNA barcode technology (Smith et al., 2008; Kress et al., 2015), restriction fragment length polymorphisms (RFLPs) (Tan et al., 2001), and single nucleotide polymorphisms (SNPs) (Lü et al., 2013). All of these methods are based on gene flow between populations. They require several generations of insects to complete, and they cannot trace the sources of an individual insect within a short time interval. The technology of 16S rDNA gene sequencing is useful for microbial identification, but it has not been used for comparing the microbial communities of different geographic populations of the CPB. In this study, we used 16S rDNA gene sequencing to evaluate the geographical variation of CPB reflected by its bacterial communities. Microbial diversity analysis methods were screened and examined for their potential to provide new ideas for using geographical origin methods. This study helps to illustrate the mechanisms of CPB invasion and adaptation by revealing the microbial community differences in different regions.

## Materials and Methods

### Samples Preparation

CPB adults were collected from Wenquan County, Chabuchar County, Jimunai County, and Urumqi of the Xinjiang Uygur Autonomous Region (XJ), and Suifenhe City of Heilongjiang Province (HL), China. All adults were collected in potato field. These five sources were taken as five samples. Furthermore, In Tacheng City of XJ, CPB beetles were collected in two areas 1 km apart and they were regarded as two samples. In Xinyuan County of XJ, CPB from different host plants (potato and eggplant) in the same area were taken as two samples. There were nine samples in total. Detailed information related to each sample is shown in Table 1. At least 20 live CPBs of each sample were collected. Each samples was split into six subsamples of two beetles each. The subsamples were used for sequencing as described below.

**TABLE 1:** Collection and sequencing of samples information of *L. decemlineata.*

Major regions	Specific sources	Sample code	Number of collected adults	Collection year/month
Xinjiang Uygur Autonomous Region	Wenquan	WQ	67	2018/07
	Jimunai	JM	73	2018/07
	Urumqi	WL	63	2018/07
	Chabuchar County	CX	76	2018/07
	Tacheng (potato)	TC	68	2018/07
	Tacheng (eggplant)	TCQ	65	2018/07
	XinyuanI	XY I	70	2018/07
	XinyuanII	XY II	77	2018/07
Heilongjiang Province	Suifenhe	HL	79	2018/07

### DNA Extraction, 16S rDNA Gene Amplification, and Sequencing

After the body surface was sterilized by 75% ethanol, two CPB adults from each source were pooled and subjected to genomic DNA extraction with the Fast DNA Stool Mini Kit (Qiagen). DNA quantity and quality were determined on 1% agarose gel. They were measured with a Nanodrop, and this was used as a template for PCR amplification. A total of 2 μg genomic DNA was used to amplify the 16S V3-V4 fragments using the primers (341F: 5′-CCTAYGGGRBGCASCAG-3′ and 806R: 5′-GGACTACNNGGGTATCTAAT-3′) by RT-PCR. The RT-PCR conditions included a denaturing step at 95°C for 5 min, followed by 20 cycles of 98°C for 20 s, 52°C for 30 s, 72°C for 30 s, and a final step of 5 min at 72°C. The PCR products were used to construct the libraries, and they were sequenced on an Ion Torrent S5^^TM^ XL platform.

### Quality Control and Taxonomy Assignment

Single-end reads were assigned to subsamples based on their unique barcode and truncated by cutting off the barcode and primer sequence. Quality filtering on the raw reads was performed under specific filtering conditions to obtain high-quality clean reads according to the Cut adapt (V1.9.1) quality control process (Martin, 2011). The reads were compared with a reference database (Silva database) using the UCHIME algorithm to detect chimera sequences, and the chimera sequences were removed (Edgar et al., 2011; Haas et al., 2011; Quast et al., 2013). The clean reads were then obtained.

### Phylogenetics

To study the phylogenetic relationships of different OTUs and the differences among the dominant species in different samples, multiple sequence alignments were conducted using MUSCLE software (Version 3.8.31).

### Data Analysis and Visualization

Clean reads were assigned into a microbe taxa table applying workflow, pick_closed_reference_otus.py, in QIIME v1.91 using the Greengenes_13_5 database. The Shannon, Simpson, Chao1, and PD whole tree indices were calculated using script, alpha_diversity.py, in QIIME v1.91. Beta diversity, based on both weighted and unweighted unifrac, was calculated by QIIME v1.91. Principal Coordinate Analysis (PCoA) was performed to obtain principal coordinates and visualize the complex, multidimensional data.

The composition of the bacterial community was calculated and displayed in R v3.5.1 using relative abundance at the phylum, class, order, family, and genus level. Relative abundance was normalized by log function before performing tSNE (t-Distributed Stochastic Neighbor Embedding) analysis with the Rtsne v0.15 package. Heatmap analysis was performed with the pheatmap v1.0.10 package at the Order and Genus levels using normalized relative abundance. LEfse v1.0.8 software was used to perform lefse analysis on relative abundance. The abundance differences of the marker bacteria in each group are shown in the bar plots.

## Results

### Sample Collection and DNA Extraction

**FIGURE 1:**
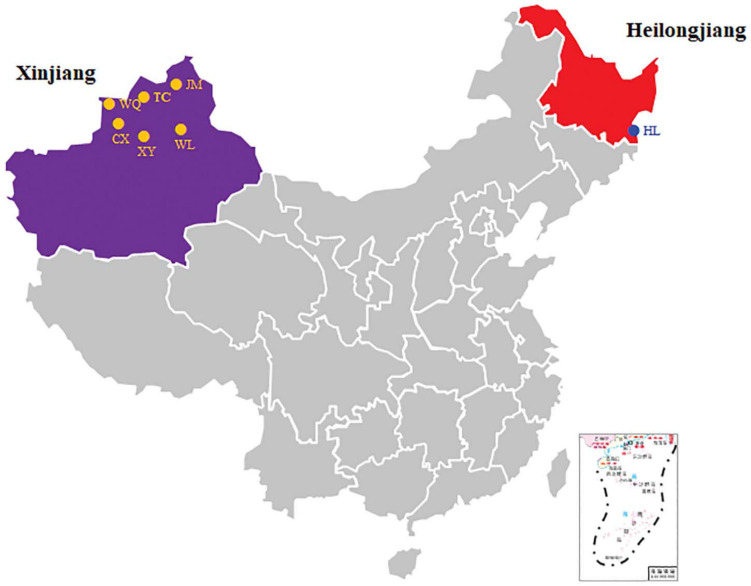
Sampling sites of CPB. The sampling sites are shown in a map of China, and the two main collection areas are indicated by different colors.

A total of 638 CPB were collected from nine sources located in the XJ and HL areas (Figure 1). The sites were Wenquan (WQ), Tacheng (potato) (TC), Tacheng (eggplant) (TCQ), Chabuchar County (CX), Xinyuan (XYI), Xinyuan (XYII), Jimunai (JM), Urumqi (WL), and Suifenhe (Heilongjiang Province, HL). The collection information and quantity of CPB were shown in Table 1. Nine samples, six times for each sample, and a total of 54 subsamples were divided and subjected to 16S rDNA sequencing.

### Sequencing and Analysis of Microbiota Diversity

**FIGURE 2:**
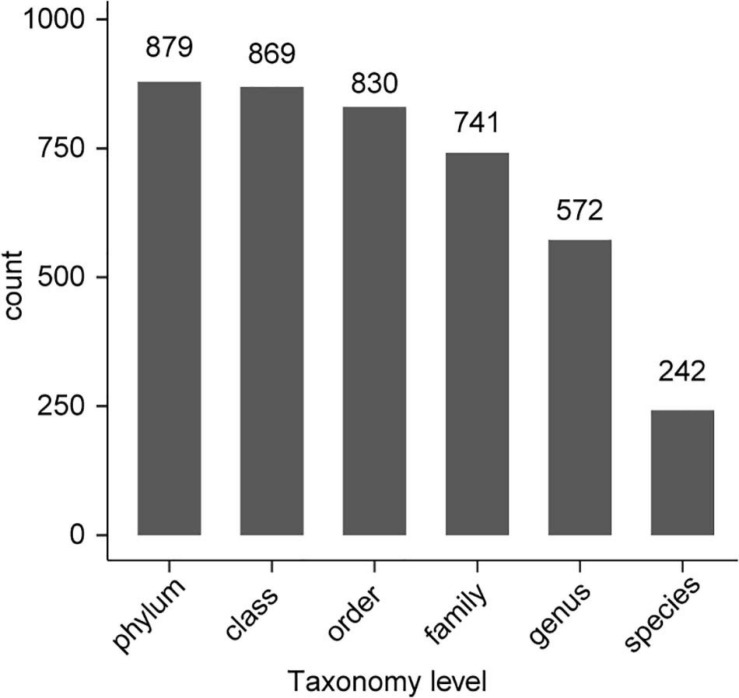
Number of microbes identified at each taxonomic level.

**FIGURE 3:**
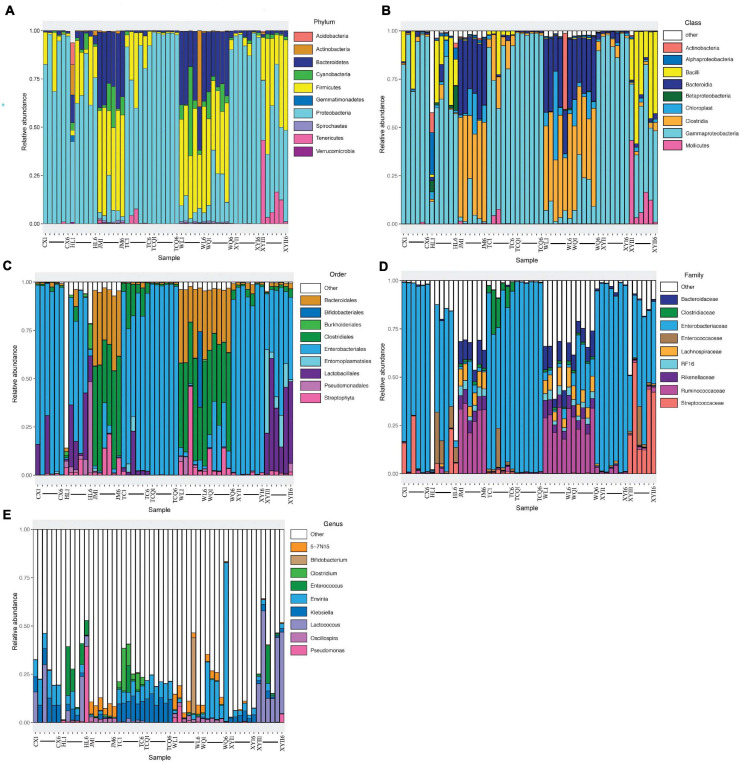
Top 10 phyla (class/order/family/genus) composition of microbiota. Each bar represents a sample, and each color represents a phylum (class/order/family/genus). Low relative abundance taxa and unclassified microbes are grouped into “Other.” **(A)** The top 10 phyla composition of microbiota. **(B)** The top 10 family composition of microbiota. **(C)** The top 10 class composition of microbiota. **(D)** The top 10 order composition of microbiota. **(E)** The top 10 genus composition of microbiota.

**FIGURE 4:**
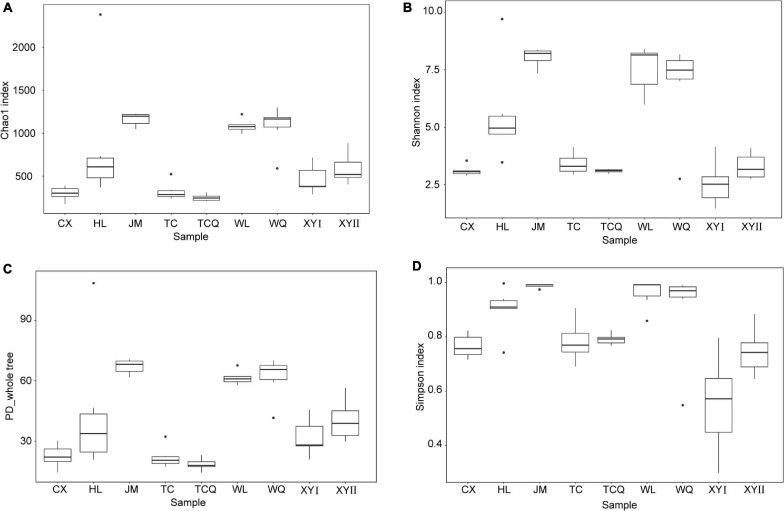
Alpha diversity indices of bacterial communities of CPB from different sampling sites. **(A)** Chao1 index. **(B)** Shannon index. **(C)** PD whole tree. **(D)** Simpson index.

All 54 subsamples were simultaneously sequenced, and they produced a total of 4,295,080 reads. All data were submitted to the National Center for Biotechnology Information (NCBI), and the BioProject number is PRJNA683770. The number of reads obtained from the *in vivo* CPB microbiota of different geographical populations differed (Table 2). Each subsamples contained 50,000−100,000 reads. After quality control, there were 4,072,777 reads. Clean data were then classified by QIIME software. We classified 879 taxa of microbes, in 34 phyla, 54 classes, 107 orders, 187 families, and 383 genera. All 879 taxa were identified to different taxonomic levels; 879 were identified to the phylum level, 869 to class, 830 to order, 741 to family, 572 to genus, and 242 to species (Figure 2). We analyzed the composition of the microbiota of 54 subsample at the phylum, class, order, family, and genus levels (Table 2 and Figure 3). The relative abundance of microorganisms was different in each sample, but there was no significant difference in the six replicates of each sample. Each source was subjected to alpha diversity comparison, and the CPB in WL, JM, and WQ had high levels of microbial diversity (Figure 4).

**TABLE 2:** Total sequencing reads statistics in six repetitions, composition and number of microorganisms in different taxonomic levels of *L. decemlineata* nine samples.

Sample	Raw reads	Clean reads	Number of OTUs	Phylum	Class	Order	Family	Genus	Species
WQ	516,775	495,204	1,225	25	41	72	116	194	118
JM	537,399	507,849	1,330	22	38	74	127	217	119
WL	520,308	494,392	1,196	22	38	68	108	173	102
CX	419,463	398,239	672	17	22	39	62	91	46
TC	423,028	400,613	672	16	22	39	58	88	40
TCQ	379,921	363,453	618	18	25	42	64	86	41
XY I	513,436	486,594	924	22	32	58	98	153	76
XY II	489,860	462,588	1,332	27	45	83	130	234	109
HL	494,890	463,845	1,252	30	43	89	150	280	152
Total	4,295,080	4,072,777	9,221	34	54	107	187	383	238

To better compare the nine samples from different sources, the microbial diversity of five taxonomic levels was analyzed to explore the difference of the same insect in different sources (Table 3). The main microorganisms at each taxonomic level were briefly introduced. At the taxonomic level of phylum, the 16S rDNA gene sequences of gut microorganisms were annotated to 34 phyla, including Proteobacteria, Firmicutes, Oxyphotobacteria, Bacteroidetes, Tenericutes, Acfinobacteria, Acidobacteria, Monospora, Chloroflexi, and Spirochaetes. A total of 54 classes are annotated, mainly including γ-Proteobacteria, Bacilli, Class Clostridia, Bacteroidetes, Mollicutes, α-Proteobacteria, and Acidimicrobiia. At the taxonomic level of order, 107 orders of gut microorganisms were annotated, mainly including Enterobacterales, Lactobacillales, Clostridiales, Pseudomonadales, Bacteroidales, Entomoplasmatales, Bifidobacteriales, and Sphingobacteriales. A total of 187 families were annotated, including Enterobacteriaceae, Streptococcidae, Spiroplamataceae, Pseudomonadaceae, Bifidobacteriaceae, Verrucomicrobiaceae, Enterococcaceae, and Rikenellaceae.

**TABLE 3:** The major microorganisms (MM) and their relative abundance (RA) of five taxonomic levels of *L. decemlineata* nine samples.

Sample	Phylum		Class		Order		Family		Genus	
	MM	RA(%)	MM	RA(%)	MM	RA(%)	MM	RA(%)	MM	RA(%)
WQ	Firmicutes	34.	Class Clostridia	32.6	Clostridiales	32.5	Enterobacteriaceae	30.4	*Pantoea*	27.1
	Proteobacteria	31.	γ-Proteobacteria	30.8	Enterobacterales	30.4	Verrucomicrobiaceae	22.1	*Oxyphotobacteria*	6.4
	Bacteroidetes	25.8	Bacteroidetes	25.8	Bacteroidales	25.8	Rikenellaceae	10.9	Others	> 60
	Oxyphotobacteria	5.4								
JM	Firmicutes	46.1	Class Clostridia	43.9	Clostridiales	43.8	Verrucomicrobiaceae	29.6%	*Oxyphotobacteria*	8.5
	Bacteroidetes Oxyphotobacteria Proteobacteria	36.1 6.9 6.7	Bacteroidetes γ-Proteobacteria	36.1 5.7	Bacteroidales Enterobacterales	35.9 5.2	Rikenellaceae Enterobacteriaceae	15.9% 5.2%	*Others*	> 90
WL	Firmicutes Bacteroidetes Oxyphotobacteria Proteobacteria	42.3 33.4 9.3 5.9	Class Clostridia Bacteroidetes γ-Proteobacteria	40.5 33.4 5.2	Clostridiales Bacteroidales Enterobacterales	40.4 33.4 2.6	Verrucomicrobiaceae Rikenellaceae Enterobacteriaceae Bifidobacteriaceae	27.2% 15.1% 2.6% 5.9%	*Oxyphotobacteria Bifidobacterium*	9.3 5.9
CX	Proteobacteria Firmicutes Bacteroidetes	> 80 1.1–8.7 0.8–2.6	γ-Proteobacteria Bacilli Class Clostridia	80.5–97.7 0.03–7.3 1.1–10.9	Enterobacterales	80.5–97.7	Enterobacteriaceae Others	80.5–97.7 < 1	*Lelliottia Enterobacter Lactococcus Pantoea*	14.4 17.1 7.3 2.3
TC	Proteobacteria	> 80	γ-Proteobacteria	80.5–97.7	Enterobacterales	80.5–97.7	Enterobacteriaceae	80.5–97.7	*Enterobacter*	9.2
	Firmicutes Bacteroidetes	1.1–8.7 0.8–2.6	Bacilli Class Clostridia	0.03–7.3 1.1–10.9			Others	< 1	*Lelliottia Enterococcus*	4.6 3.9
TCQ	Proteobacteria Firmicutes Bacteroidetes	> 90 1.1–8.7 0.8–2.6	γ-Proteobacteria Bacilli Class Clostridia	80.5–97.7 0.03–7.3 1.1–10.9	Enterobacterales	80.5–97.7	Enterobacteriaceae Others	80.5–97.7 < 1	*Leliottia Enterobacter* Others	19.4 20.5 < 1
XY I	Proteobacteria Firmicutes Bacteroidetes	> 90 1.1–8.7 0.8–2.6	γ-Proteobacteria Bacilli Class Clostridia	80.5–97.7 0.03–7.3 1.1–10.9	Enterobacterales	80.5–97.7	Enterobacteriaceae Others	80.5–97.7 < 1	*Leliottia Enterobacter* Others	58.3 3.8 < 1
XY II	Proteobacteria Firmicutes Tenericutes	46.6 36.1 12.9	γ-Proteobacteria Bacilli	46.3 33.5	Enterobacterales Lactobacillales Entomoplasmatales	45.4 33.4 12.9	Enterobacteriaceae Streptococcidae Spiroplamataceae Enterococcaceae Verrucomicrobiaceae	45.4 29.6 12.9 3.8 1.7	*Lactococcus Spiroplasma Lelliottia Enterococcus*	29.6 12.9 7.4 3.8
HL	Proteobacteria Firmicutes Bacteroidetes	63.7 17.4 9.0	γ-Proteobacteria Bacilli	58.8 15.7	Enterobacterales Lactobacillales Pseudomonadales Sphingobacteriales	41.7 15.4 10.8 4.5	Enterobacteriaceae Enterococcaceae Pseudomonadaceae Streptococcidae	41.7 9.7 6.7 5.1	*Lelliottia Enterococcus Enterobacter Pseudomonas Lactococcus*	15.5 9.7 7.9 6.7 5.1

At the taxonomic level of genus, 383 genera were annotated, including *Pantoea*, *Lelliottia*, *Lactococcus*, *Spiroplasma*, *Pseudomonas*, *Bifidobacterium*, *Enterobacter*, and *Enterococcus*. Eight were from XJ and one was from HL among the nine samples. The dominant bacteria at the genus level were compared between XJ and HL. Pseudomonas was the unique dominant microorganism in HL. Four microorganisms (*Lelliottia*, *Enterococcus*, *Enterobacter*, and *Lactococcus*) were common within the two regions. At the family level, Enterobacteriaceae was dominant in all samples, and in many of the eight samples, the dominant species were the same with different relative abundance.

### Verification of tSNE Method’s Ability to Distinguish Geographical Source

**FIGURE 5:**
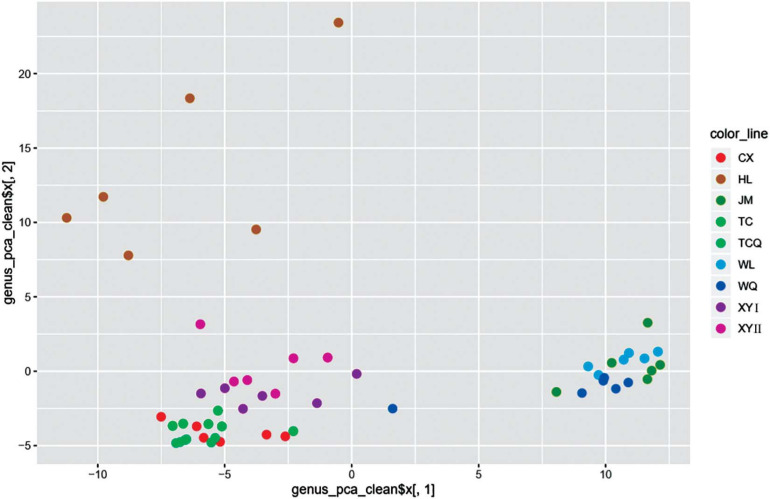
PCA of the bacterial community of CPB collected from nine areas.

**FIGURE 6:**
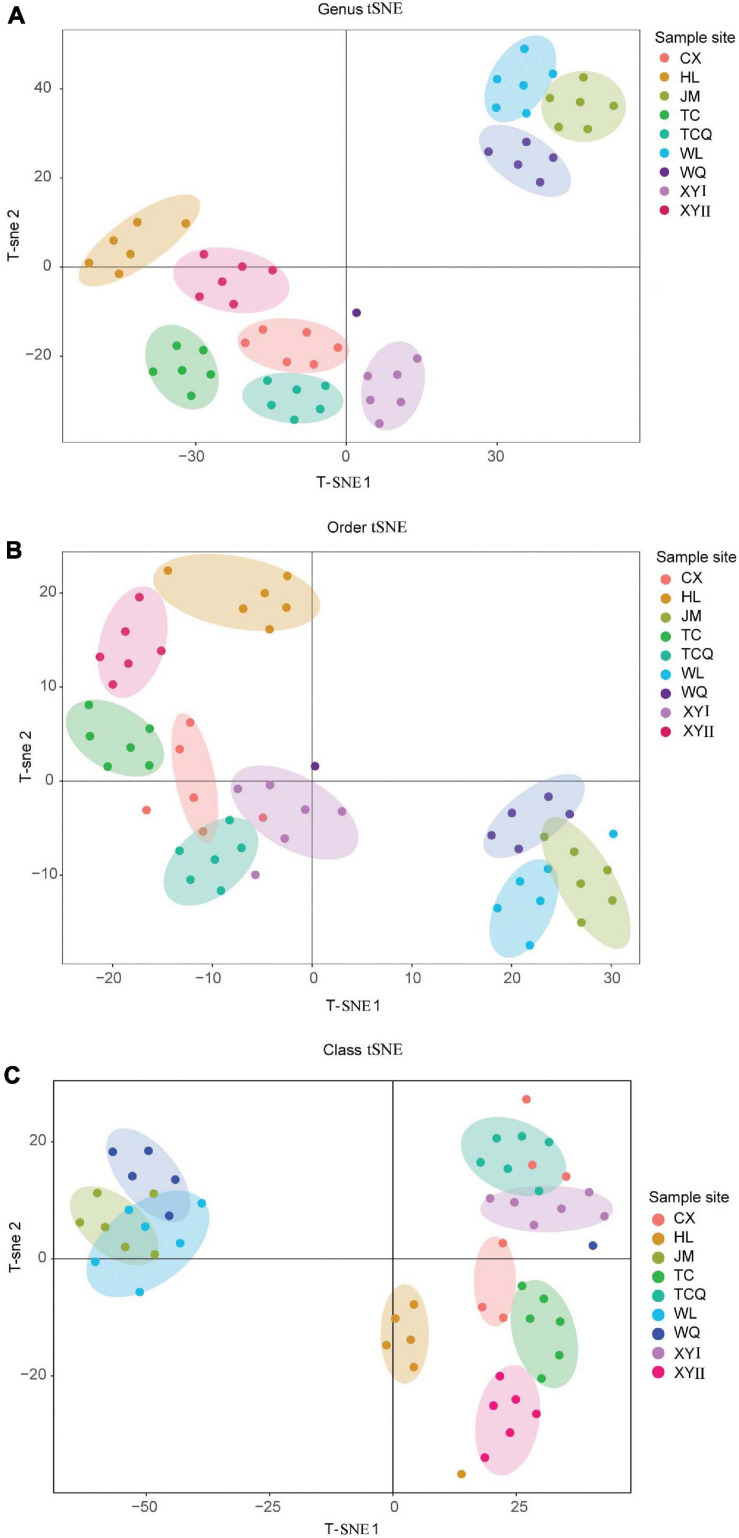
TSNE analysis of CPB collected from different areas. Each point represents a sample, and each color represents a sample site. **(A)** TSNE analysis at the genus level. **(B)** TSNE analysis at the order level. **(C)** TSNE analysis at the class level.

To study the relationship between microbial diversity and geographic source, we analyzed microbiota data using Principal Component Analysis (PCA). The dots that represent CPB from the nine areas did not adequately distinguish geographical source (Figure 5). Thus, we used a non-linear algorithm tSNE. This algorithm is based on a probability distribution of random walks on the neighborhood graph to find structures within a dataset. We performed tSNE at three taxonomic levels. CPB from the same geographical area grouped together at the class and genus levels (Figure 6). This result suggested that tSNE is better than PCA in extracting geographic factors affecting insect microbiota. The microbiota of CPB in WL, JM, and WQ were similar and clustered together with a high abundance of bacteria. The other samples had a low abundance of bacteria. Order and genus appeared to be appropriate proper taxonomic levels to distinguish geographical clusters of CPB.

**FIGURE 7:**
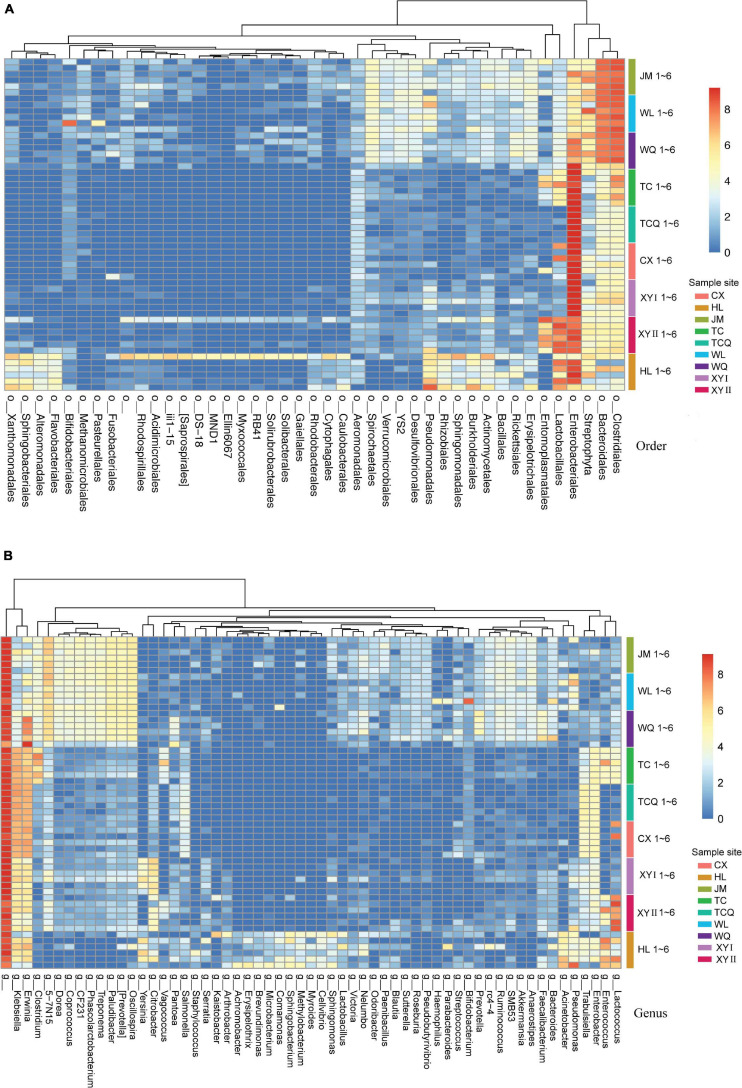
Differences in microbes at the order/genus level for CPB from different geographical sources. Low relative abundance (<0.01) and unidentified order/genus microbes were grouped into o__/g__. **(A)** Heatmap of bacterial communities of CPB from different sites (order level). **(B)** Heatmap of bacterial communities of CPB from different sites (genus level).

Since the tSNE had a better clustering effect at the order and genus levels, we performed a heat map analysis of these two levels (Figure 7). The heat map showed that the composition of the microbes of CPB in JM, WL, and WQ was similar, at the two levels. At the order level, *Clostridiales*, *Bacteroidales*, and *Streptophyta*, and at the genus level, *Paludibacter*, *Prevotella*, and *Oscillospira* in the three regions were similar. These data distinguished JM, WL, and WQ from the other six regions, and the results are similar to those of tSNE.

The tSNE method can be useful for extracting the geographical information affecting the microbiota of CPB. In tSNE cluster analysis, microbes in WL, JM, and WQ were similar and clustered together and were named group A. The remaining six sources were named group B. We compared groups A and B by Linear discriminant analysis Effect Size (LEfSe) and found significant differences in OTU richness between the groups (Supplementary Figure 1). At the order level, the abundance of Enterobacteriales, Lactobacillales, Aeromonadales, and Clostridiales in group B was significantly higher than that in group A. The abundance of Rickettsiales, Verrucomicrobiales, Desulfovibrionales, Erysipelotrichales, YS2, and Spirochaetales in group A was significantly higher than that in group B. These significant differences in microbial populations can be used as biomarkers that can divide the geographical sources of the CPB.

Samples from TC were divided into CPB feeding on potatoes and CPB feeding on eggplants. We compared the species differences between these two groups by LEfSe (Supplementary Figure 2). At the genus level, the abundances of microbial populations in TCQ and TC were significantly different; the differential microorganisms in TCQ were *Citrobacter* and *Salmonella*, and in TC these were *Dorea*, *Lactococcus*, *CF231*, *Vagococcus*, *Enterococcus*, and *Clostridium*. These microorganisms may be regarded as biomarkers to distinguish one species from different host plants within the same source. However, two samples from two plots in Xinyuan County were compared for their microbial species composition by LEfSe analysis (Supplementary Figure 3). At the genus level, the abundance of *Cronobacter* and *Yersiniavan* in XYI was significantly higher than that in XYII, and that of *Vagococcus* and *Enterococcus* in XYII was significantly higher than that in XYI. The microorganisms appear to be related to the microenvironment, like host plant, soil, temperature, humidity, and other conditions.

## Discussion

The bacterial communities of CPB from different sources in China were compared with 16S high throughput sequencing. Abundant and diverse bacteria were found in CPB. At the phylum level, Bacteroides, Firmicutes, and Proteobacteria were the most important groups. This is consistent with studies on other coleopteran endomicrobiomes (Durand et al., 2015). At the genus level, there were large differences in the bacterial communities, especially among different plots within the same area. Some bacteria were the same as the reported enteric bacteria (Michael et al., 2008; Muratoglu et al., 2011; Krawczyk et al., 2015), including *Lactococcus* and *Enterobacter*, and some were different, e.g., *Dorea*, *Vagococcus*, and *Clostridium*, suggesting that these bacteria may be secondary bacteria related to the environment. The nine samples have similar microorganisms with different relative abundance, but regional specificity was also found. For example, *Pseudomonas* was specific to HL. At higher taxonomic levels there were fewer differences. The number of five taxonomic levels and the microbial diversity also showed the differences of microbial diversity among samples from different sources (Tables 2, 3). Differences in host plant also led to differences in the microbial diversity, such as in TC and TCQ (Supplementary Figure 2). The microbial diversity showed that the gut microbes are closely related to several factors: one is the different location, the second is the different potato varieties, the third is the different temperature, humidity and other weather conditions in the field, the fourth is that the invasion source may be different, CPB of XJ may come from the northwest neighboring countries, CPB of HL may come from Russia. All of these differences may lead to differences in microorganisms.

A diversity of bacterial communities in different sources of CPB is reported here for the first time. Proteobacteria are dominant in all of the populations. Proteobacteria play an important role in digestion and nutrition in some insects. For example, γ-proteobacteria in bees can encode pectin-degrading enzymes and participate in the lysis of pollen walls (Engel et al., 2012). Firmicutes bacteria were also common in the gut tract, including Enterococcus, Streptococcus, Staphylococcus, and Lactobacillus. Firmicutes bacteria are important in mammalian material and energy metabolism (Ley et al., 2008). *Clostridium* bacteria can effectively degrade cellulose and hemicellulose to produce amino acids. Enterococcus in the gut of gypsy moths can help resist pathogen invasion (Chen et al., 2016).

The diversity of CPB microorganisms from different sources further illustrates the role of microorganisms in invasion and adaptation to local conditions, and it is possible to use 16S sequencing technology combined with the tSNE method to distinguish geographical populations. This is the first example of comparing CPB population sources using the bacterial community, although many factors, such as different developmental status and sex can affect the microbial composition (Guo et al., 2015; Kuan et al., 2015). We have provided a potential method for establishing the geographical source of an organism based on bacterial community factors rather than common genetic methods (Abdel-Aziem et al., 2005; Virgilio et al., 2012; Jung et al., 2016; Kang et al., 2019). The applicability of the bacterial method needs verification in other insects and the sequencing, data analysis and related methods need to be improved.

## Data Availability Statement

The datasets presented in this study can be found in online repositories. The names of the repository/repositories and accession number(s) can be found below: https://www.ncbi.nlm.nih.gov/, PRJNA683770.

## Author Contributions

YY and YW designed and conceived the study. XY assisted in the collection of CPB sources. HL assisted in the analyses of sequencing data. YY, WS, and JZ developed the project and revised the manuscript. All authors contributed to scientific discussions and manuscript preparation.

## Conflict of Interest

YW was employed by company Biowavelet Ltd. The remaining authors declare that the research was conducted in the absence of any commercial or financial relationships that could be construed as a potential conflict of interest.

## Supplementary Material

Supplementary Figure 1 LEfse analysis showing biomarkers for Cluster A and Cluster B. The bar plot shows the relative abundance difference of these biomarkers between the two clusters. (Cluster A contains JM, WL, and WQ. Cluster B contains TC, TCQ, CX, XYI, XYII, and HL.) **(A−J)** Relative abundance of each biomarker in Cluster A and Cluster B.

Supplementary Figure 2 LEfse analysis showing the biomarkers between TC and TCQ. The bar plot shows the relative abundance difference for each biomarker between TC and TCQ. **(A–H)** Relative abundance of each biomarker in TC and TCQ.

Supplementary Figure 3 LEfse analysis for XYI and XYII. The bar plot shows the difference in relative abundance for each biomarker identified by Lefse between XYI and XYII. **(A–D)** Relative abundance of each biomarker in XYI and XYII.
