# Supplementary figures and images for: Comparison of Microbial Communities in Colorado Potato Beetles (Leptinotarsa decemlineata Say) Collected From Different Sources in China
Source: Front Microbiol. 2021 Mar 19;12:639913. doi: 10.3389/fmicb.2021.639913 (PMC8017321; doi:10.3389/fmicb.2021.639913)

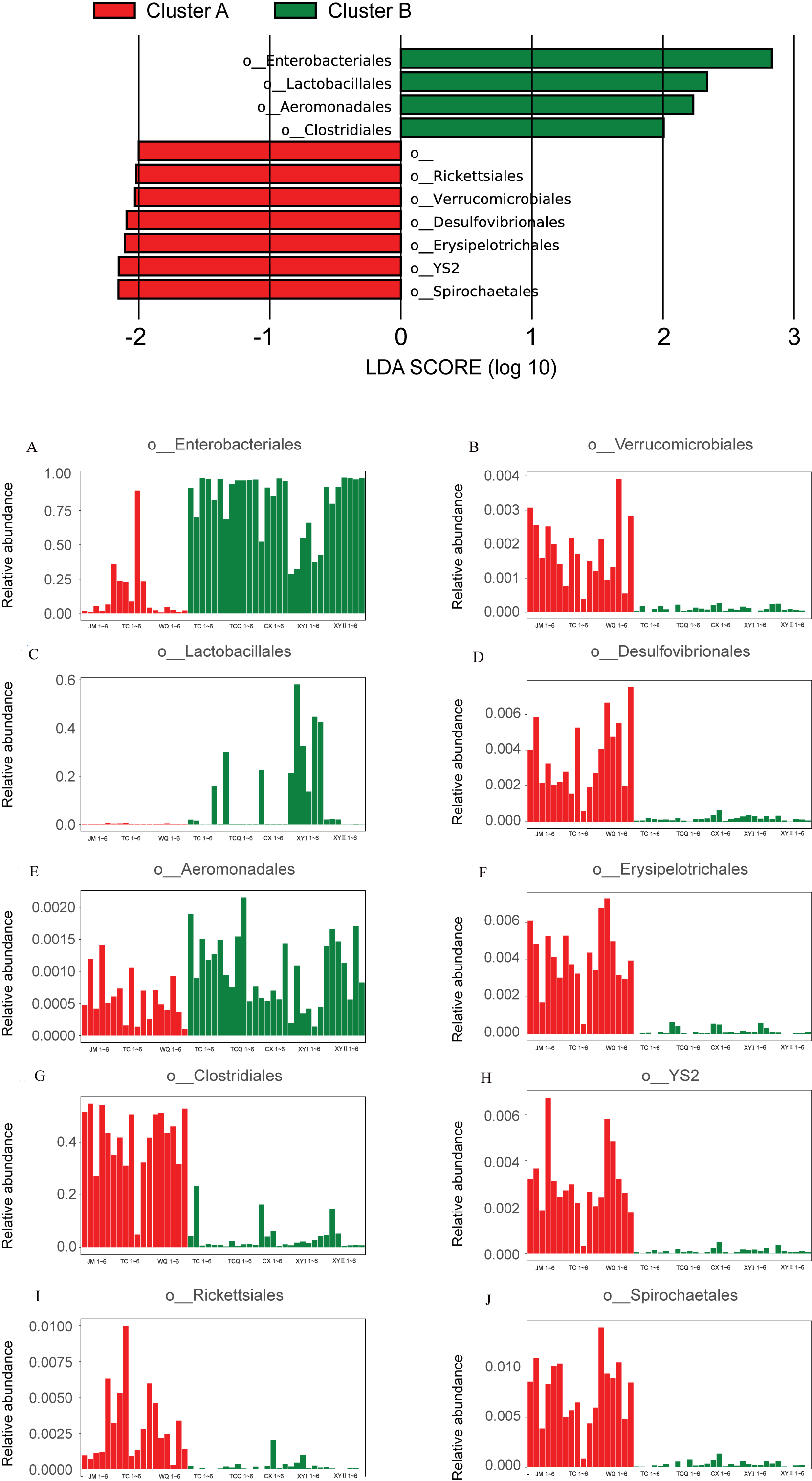

Supplement: Supplementary Figure 1 — LEfse analysis showing biomarkers for Cluster A and Cluster B. The bar plot shows the relative abundance difference of these biomarkers between the two clusters. (Cluster A contains JM, WL, and WQ. Cluster B contains TC, TCQ, CX, XYI, XYII, and HL.) (A−J) Relative abundance of each biomarker in Cluster A and Cluster B. [file Image_1.TIFF]

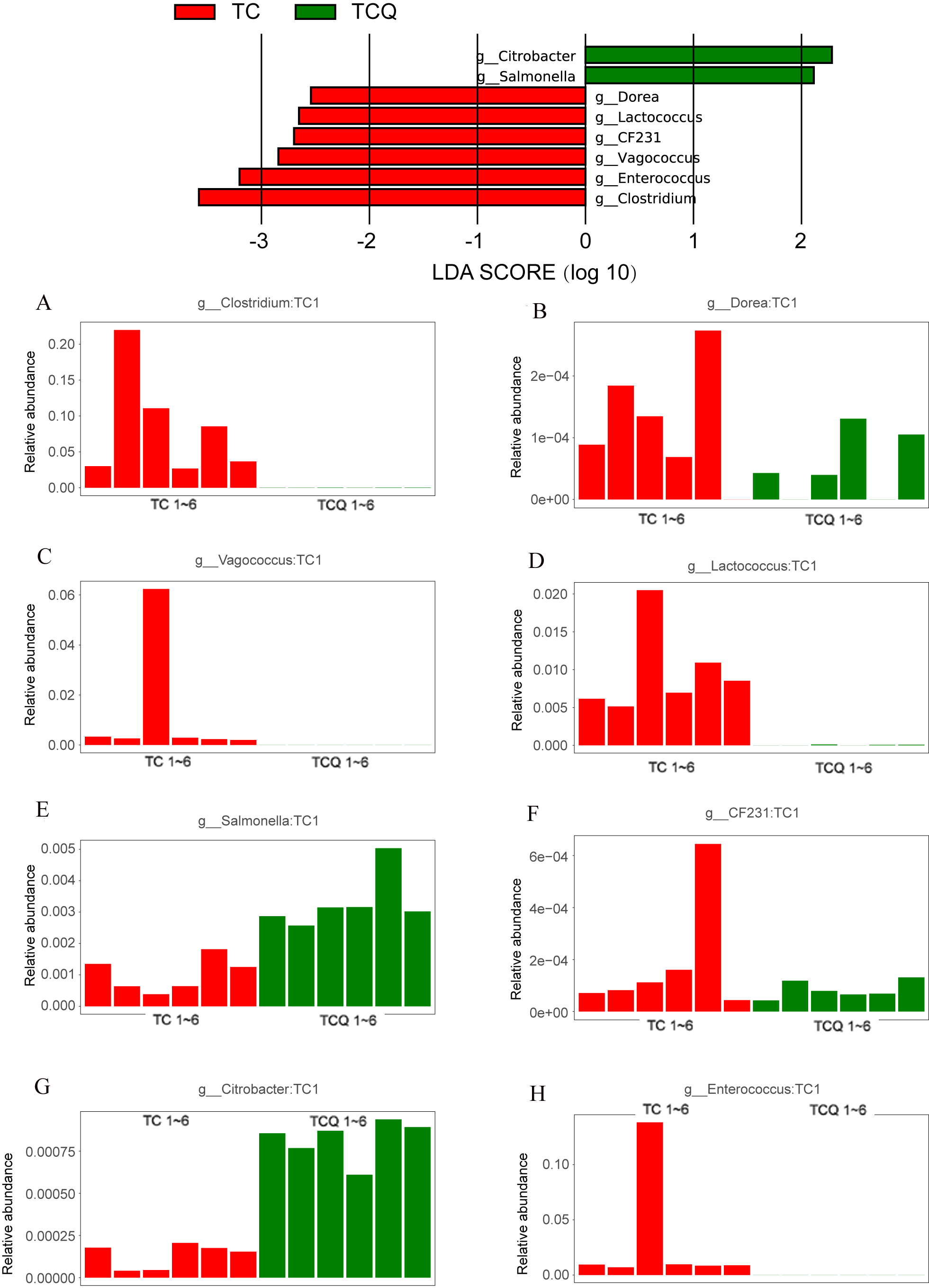

Supplement: Supplementary Figure 2 — LEfse analysis showing the biomarkers between TC and TCQ. The bar plot shows the relative abundance difference for each biomarker between TC and TCQ. (A–H) Relative abundance of each biomarker in TC and TCQ. [file Image_2.TIFF]

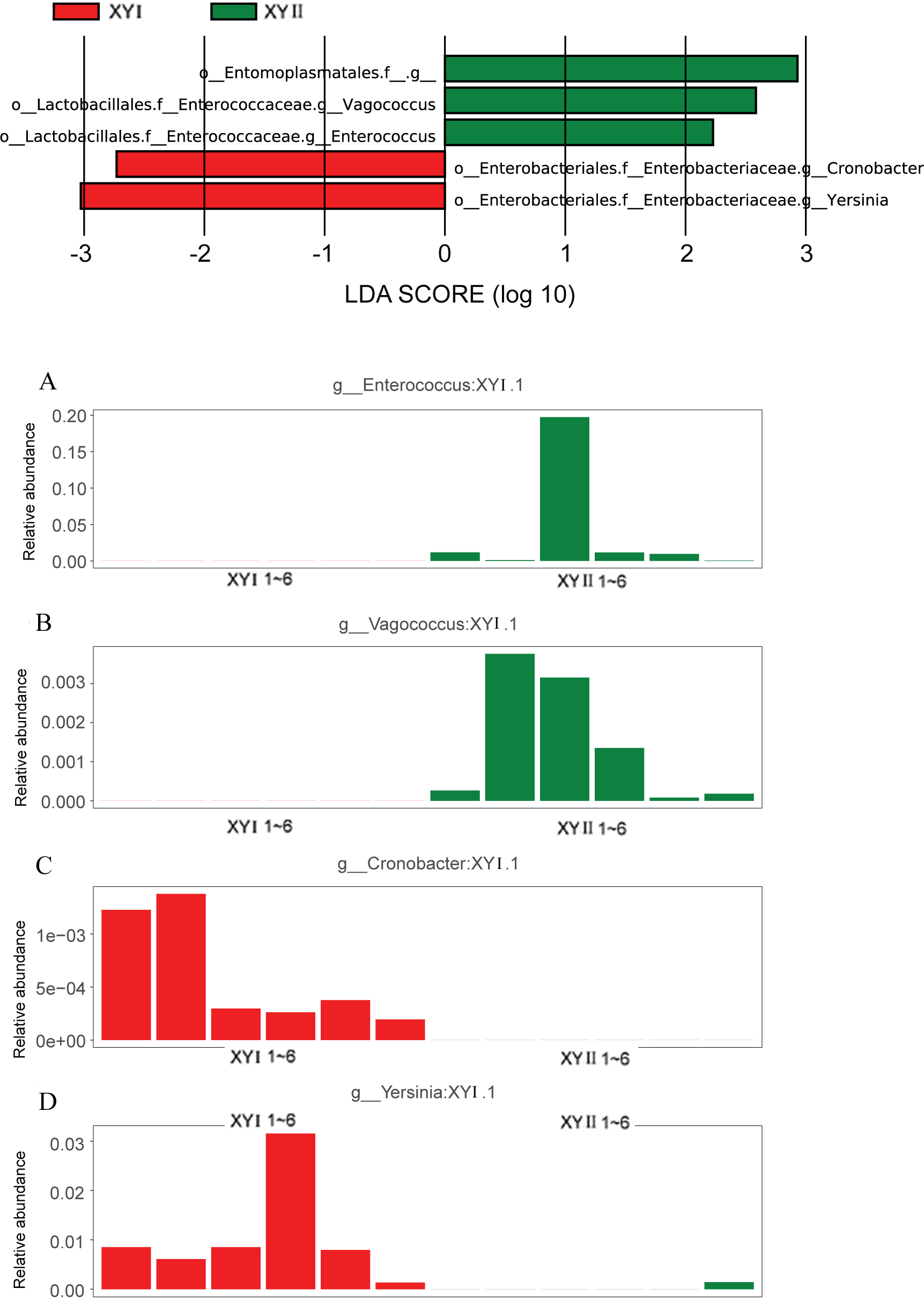

Supplement: Supplementary Figure 3 — LEfse analysis for XYI and XYII. The bar plot shows the difference in relative abundance for each biomarker identified by Lefse between XYI and XYII. (A–D) Relative abundance of each biomarker in XYI and XYII. [file Image_3.TIFF]
